# Effects of Chronic Sleep Restriction on the Brain Functional Network, as Revealed by Graph Theory

**DOI:** 10.3389/fnins.2019.01087

**Published:** 2019-10-11

**Authors:** Farzad V. Farahani, Magdalena Fafrowicz, Waldemar Karwowski, Pamela K. Douglas, Aleksandra Domagalik, Ewa Beldzik, Halszka Oginska, Tadeusz Marek

**Affiliations:** ^1^Computational Neuroergonomics Laboratory, Department of Industrial Engineering & Management Systems, University of Central Florida, Orlando, FL, United States; ^2^Department of Cognitive Neuroscience and Neuroergonomics, Institute of Applied Psychology, Jagiellonian University, Kraków, Poland; ^3^Institute for Simulation and Training, University of Central Florida, Orlando, FL, United States; ^4^Department of Psychiatry and Biobehavioral Sciences, University of California, Los Angeles, Los Angeles, CA, United States; ^5^Brain Imaging Core Facility, Malopolska Centre of Biotechnology, Jagiellonian University, Kraków, Poland

**Keywords:** sleep restriction, functional connectivity, fMRI, graph theory, small-world, connectome, brain networks

## Abstract

Sleep is a complex and dynamic process for maintaining homeostasis, and a lack of sleep can disrupt whole-body functioning. No organ is as vulnerable to the loss of sleep as the brain. Accordingly, we examined a set of task-based functional magnetic resonance imaging (fMRI) data by using graph theory to assess brain topological changes in subjects in a state of chronic sleep restriction, and then identified diurnal variability in the graph-theoretic measures. Task-based fMRI data were collected in a 1.5T MR scanner from the same participants on two days: after a week of fully restorative sleep and after a week with 35% sleep curtailment. Each day included four scanning sessions throughout the day (at approximately 10:00 AM, 2:00 PM, 6:00 PM, and 10:00 PM). A modified spatial cueing task was applied to evaluate sustained attention. After sleep restriction, the characteristic path length significantly increased at all measurement times, and small-worldness significantly decreased. Assortativity, a measure of network fault tolerance, diminished over the course of the day in both conditions. Local graph measures were altered primarily across the limbic system (particularly in the hippocampus, parahippocampal gyrus, and amygdala), default mode network, and visual network.

## Introduction

Humans spend roughly one-third of their lifetime sleeping. Although sleep has been a subject of research for hundreds of years, the neurobiological underpinnings of sleep, particularly sleep deprivation, remain somewhat elusive (Kaufmann et al., 2016). Sleep deficit leads to various health conditions such as cardiovascular diseases, obesity, diabetes, immune system dysfunction, and many cognitive and emotional impairments (Rogers, 2003; Goel et al., 2009; Gamaldo et al., 2012; Tobaldini et al., 2017; Reutrakul and Van Cauter, 2018). Although a lack of sleep can damage all organs in the human body, its impact on the central nervous system appears to be the most severe (Cirelli and Tononi, 2008). Based on neuroimaging studies, the consequences of sleep loss on negative and positive emotion, memory and attention, and hippocampal learning have become subjects of great interest to many researchers (Krause et al., 2017).

Sleep deprivation triggers mood alterations in negative emotional appraisal, including irritability, emotional volatility, aggression, anxiety (Dinges et al., 1997; Anderson and Platten, 2011; Kamphuis et al., 2012; Minkel et al., 2012), and suicidal ideation and behaviors (Joiner, 2007; Stubbs et al., 2016). The emotional effects of sleep deficit are associated with not only enhanced reactivity toward negative stimuli but also altered response patterns to pleasure-evoking stimuli (Gujar et al., 2011). Furthermore, adequate sleep is crucial for memory consolidation (Walker and Stickgold, 2004), and sleep plays an essential role in preparing the brain for the formation of new memories (Yoo et al., 2007). A single night of sleep loss can affect the hippocampal performance in encoding episodic memory, an effect associated with altered patterns of connectivity in alertness networks of the thalamus and brainstem (Yoo et al., 2007). Apart from its effect on emotional and memory functions, a lack of sleep strongly affects vigilant attention (Lim and Dinges, 2008). In this regard, the vulnerability of the attention and salience networks to acute total sleep deprivation has been identified in the form of activity reduction and functional connectivity alterations within these networks (Ma et al., 2015).

Functional MRI (fMRI) is a powerful non-invasive imaging modality that measures hemodynamic fluctuations as a proxy for neural activity (Logothetis, 2002). A better understanding of the neurophysiological mechanisms underlying sleep-related abnormalities can be obtained by identifying functional connectivity alterations. fMRI studies on sleep deprivation have primarily focused on resting-state data. For example, Shao et al. (2014) have reported diminished resting-state functional connectivity between the amygdala and executive control areas (e.g., dorsolateral prefrontal, anterior cingulate, and inferior frontal) after 36 h of total sleep deprivation (Shao et al., 2014). Increased functional connectivity between the dorsal nexus and the dorsolateral prefrontal cortex has been demonstrated in another experiment (Bosch et al., 2013) in which participants slept from 3:06 AM (±1:36 h) until 6:48 AM (±2:48 h). Moreover, sleep deprivation leads to decreased connectivity profiles within the default mode network (DMN), and reduced anticorrelated activity between the DMN and task-positive network, during both the resting state and visual attention tasks. These findings suggest that highly integrated (or segregated) brain regions become less integrated (or segregated) throughout sleep loss (Sämann et al., 2010; De Havas et al., 2012; Yeo et al., 2015).

Graph-based network analysis provides a framework that can be used to capture the topological organization of the human brain connectome through the computation of a series of local and global features such as small-worldness, clustering coefficient, characteristic path length, modular structure, degree centrality, and assortativity of brain networks (Bullmore and Sporns, 2009, 2012; He and Evans, 2010; Meunier et al., 2010; Rubinov and Sporns, 2010; van den Heuvel and Sporns, 2013; Farahani et al., 2019). The small-world paradigm is of particular interest in characterizing human brain organization, because it supports efficient information segregation and integration across brain regions with low energy and wiring costs, and it is appropriate for examining complex brain dynamics (Watts and Strogatz, 1998). Recent studies have shown that local and global measures of brain networks undergo topological changes under different neurological disorders (Xia and He, 2011; Fornito et al., 2012; Filippi et al., 2013; Dai and He, 2014; Stam, 2014; Fornito and Bullmore, 2015; Gong and He, 2015; Abós et al., 2017; Fleischer et al., 2017; Hojjati et al., 2017; Jalili, 2017; Miri Ashtiani et al., 2018).

Most previous studies that have applied graph theory to track alterations in brain activity are based on resting-state fMRI (Farahani et al., 2019). However, some studies suggest that certain connectivity properties can be identified only by examining brain topology during task performance (Pezawas et al., 2005; Bilek et al., 2013). In this paper, we used task-based fMRI data to study functional connectivity alterations after sleep restriction. To this end, we investigated the topological changes in brain functional connectivity patterns induced by a spatial cueing task to evaluate participants’ sustained attention in both rested wakefulness and chronic sleep restriction. In general, the purpose of the current study was to investigate global and local changes in the network topology between rested wakefulness and sleep restriction conditions, as well as the diurnal variability across both conditions. The findings might provide potential imaging markers of sustained attention impairment induced by chronic sleep deficit, as well as daily variability.

## Materials and Methods

### Experiment Protocol

Thirteen healthy female participants (mean age 23.4 ± 2.0 years) completed this study. All subjects met the experiment requirements, including right-handedness, right-eye dominance, normal or corrected-to-normal visual acuity, and an absence of physical, psychiatric, and sleep-related disorders. None of them showed an elevated level of daytime sleepiness, as controlled with Epworth Sleepiness Scale (Johns, 1991), nor sleep problems controlled using Pittsburgh Sleep Quality Index (Buysse et al., 1989). They were remunerated for their participation. Written informed consent was obtained from all participants before the study, which was approved by the Bioethics Committee at the Jagiellonian University, Poland. The reason why females are selected for the study is because of the known gender difference in sleep need and sleep deficit consequences which are greater in women than in men (Ferrara and De Gennaro, 2001; Oginska and Pokorski, 2006).

**FIGURE 1:**
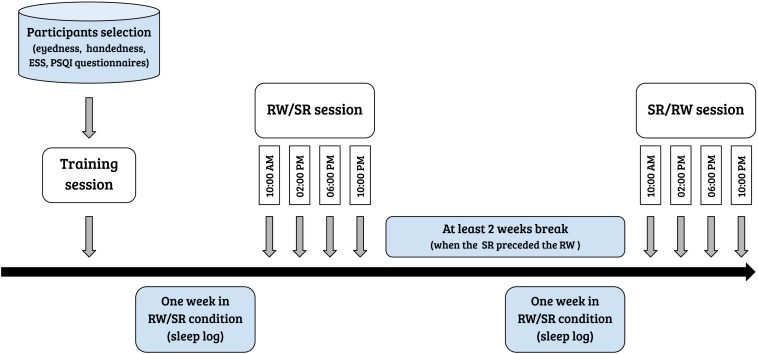
Flowchart of the steps of the experiment.

Participants completed the study in three visits: (1) a training session, (2) a session after a week with unrestricted, fully restorative sleep, denoted the rested wakefulness (RW) session, and (3) a session after a week of sleep curtailment by 35%, denoted the sleep restriction (SR) session. The average sleep during RW and SR conditions were 505 min (8 h 25 min) ± 63 min, and 310 min (5 h 10 min) ± 58 min, respectively. The order of experimental sessions (i.e., RW session and chronic SR session) was counterbalanced across all participants. The sessions were separated by at least 2 weeks to minimize the residual effects of sleep curtailment on cognitive performance when a chronic SR session preceded the RW session. Each experiment contained 13 consecutive runs with 46 trials each in both the RW and SR conditions. One day before the first experimental day, the participants were extensively trained on the experimental task to avoid the influence of a learning process on performance. Figure 1 displays the steps of the experiment in chronological order. All participants performed the experimental tasks four times during the day, at 10:00 AM, 2:00 PM, 6:00 PM, and 10:00 PM (in both sessions). The subjects spent the experimental days in a controlled laboratory environment, and a semi-constant routine protocol was applied, in which the room temperature and light intensity were kept constant. During experimental days, participants were allowed to engage in non-strenuous activities (e.g., reading, watching a video, and conversation). Research assistants observed the participants and prevented them from napping via verbal reminders. The participants’ diets during the experimental days were controlled to avoid caffeine or tryptophan intake. Alcohol consumption during the preceding week and the experimental days was not allowed.

### Experiment Task

A modified spatial cueing task (Posner, 1980) was used to evaluate participants’ sustained attention in both the RW and the chronic SR conditions. Stimuli generated by red laser diodes were presented on the horizontal axis on a panel integrated with a saccadometer system attached to each subject’s head approximately 3 cm from the eyes. There were five diodes on the panel: a central diode for fixation, and left and right diodes at 1° and 10° visual angles for cue stimuli and target, respectively. A calibration procedure before each session was conducted in which the participants looked three times at each stimulus for a 1 s period. The participants were instructed to direct their attention and gaze from the fixation point to targets only if they were preceded by a cue.

**FIGURE 2:**
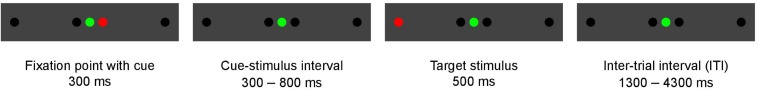
Illustration of a single experimental trial. This trial shows an incongruent task, because the cue and target are presented in opposite directions.

Each experimental trial started with a fixation point presented with a green laser diode at the center of the panel screen (Figure 2). Simultaneously, a cue was presented with a red laser diode at 1° to the right or left of the fixation point for 300 ms. After 300 ms to 800 ms (varying in steps of 100 ms), a target stimulus flashed for 500 ms at 10° to the right or left of the fixation point, and this was followed by an inter-trial interval of 1300 to 4300 ms (varying in steps of 500 ms). Then a new trial began immediately. To improve the sampling rate of the hemodynamic response, the phase of the target was varied relative to the image acquisition (Josephs et al., 1997; Toni et al., 1999), thus resulting in the final temporal resolution of 100 ms. The task comprised stimuli with cues congruent to the target (58%), stimuli with cues incongruent to the target (15%), and stimuli without a cue (27%). The trial sequence was pseudo-randomized to counterbalance the presentation of each trial type. A total of 598 task stimuli were included in each measurement. The task lasted approximately 42 min. Every participant was exposed to the same order of stimuli with the same timing, however, the trial order differed between sessions.

### Data Acquisition

#### Eye-Tracking Data

Eye movements were monitored using a Saccadometer Research MRI system, and then analyzed using Research Analyzer software (Ober-Consulting, Poland). The saccadometer system measures right eye movement in horizontal axis using direct near-infrared technology. It has 500 Hz sampling frequency, measuring range ±20° of visual angle and average spatial resolution of 15′. Saccades were detected with the use of a velocity criterion - eye movements faster than 5°/s.

#### Functional MRI Data

Magnetic resonance imaging was performed with a 1.5T Signa HDxt MRI scanner (GE Healthcare Systems, Milwaukee, United States). High-resolution whole-brain anatomical images were acquired with T_1_-weighted multi-echo volumetric MRI, and a total of 60 axial slices were obtained (matrix size = 512 × 512; time repetition TR = 25.0 s; time echo TE = 6.0 ms; field of view FOV = 22 × 22 cm^2^; flip angle = 45°). Blood-oxygenation-level dependent (BOLD) functional scans were acquired with a T_2_^∗^-weighted EPI pules sequence (matrix size = 128 × 128; TR = 3.0 s; TE = 60 ms; flip angle = 90°). Each whole-brain image was covered with 20 axial slices taken in an interleaved fashion.

### Graph Analysis and Computation

**FIGURE 3:**
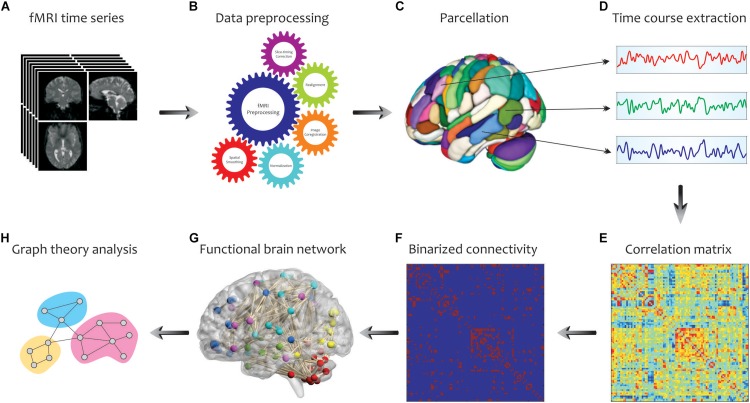
Schematic representation of brain network construction and graph theoretical analysis using fMRI data. After processing **(B)** of the raw fMRI data **(A)** and division of the brain into different parcels **(C)**, several time courses were extracted from each region **(D)** to create the correlation matrix **(E)**. To reduce the complexity and enhance visual understanding, the binary correlation matrix **(F)** and the corresponding functional brain network **(G)** were constructed, respectively. Eventually, by quantifying a set of topological measures, graph analysis was performed on the brain’s connectivity network **(H)**. Adapted from Farahani et al. (2019).

By considering the human brain as a large-scale and complex network, graph-based methods help to analyze the human connectome by providing a mathematical representation of pairwise relations between brain regions of interest (ROIs). An overview of our analysis pipeline is shown in Figure 3. First, fMRI data were collected for all subjects and underwent standard pre-processing with the SPM12 package^1^, which included slice timing correction, realignment, image coregistration, normalization based on segmentation, and spatial smoothing. Notably, we did not regress out the task effects from the regional time courses, because the task-based activities were superior to the resting-state spontaneous activities, and certain connectivity profiles during task execution might not be attainable at rest. The data were then aligned to an automated anatomical labeling (AAL) atlas, which was used to define ROIs (i.e., graph nodes) for brain network construction. The AAL atlas parcels the entire brain into 116 distinct anatomical units (Tzourio-Mazoyer et al., 2002), including 90 cortical and subcortical areas (regions 1–90) as well as 26 cerebellar areas (regions 91–116). The representative time course of each region was then extracted by averaging BOLD signals across all voxels in the region. Then, Pearson’s correlation coefficients were computed between time series from all pairs of regions, followed by converting them into *z* values using Fisher’s r-to-z transformation to correct for non-normality. This step yielded a symmetric correlation matrix *C*_*ij*_ (size 116 × 116) for each subject, whose element in the *i*, *j* position was the linear correlation between time courses of regions *i* and *j* (i.e., graph edges).

**FIGURE 4:**
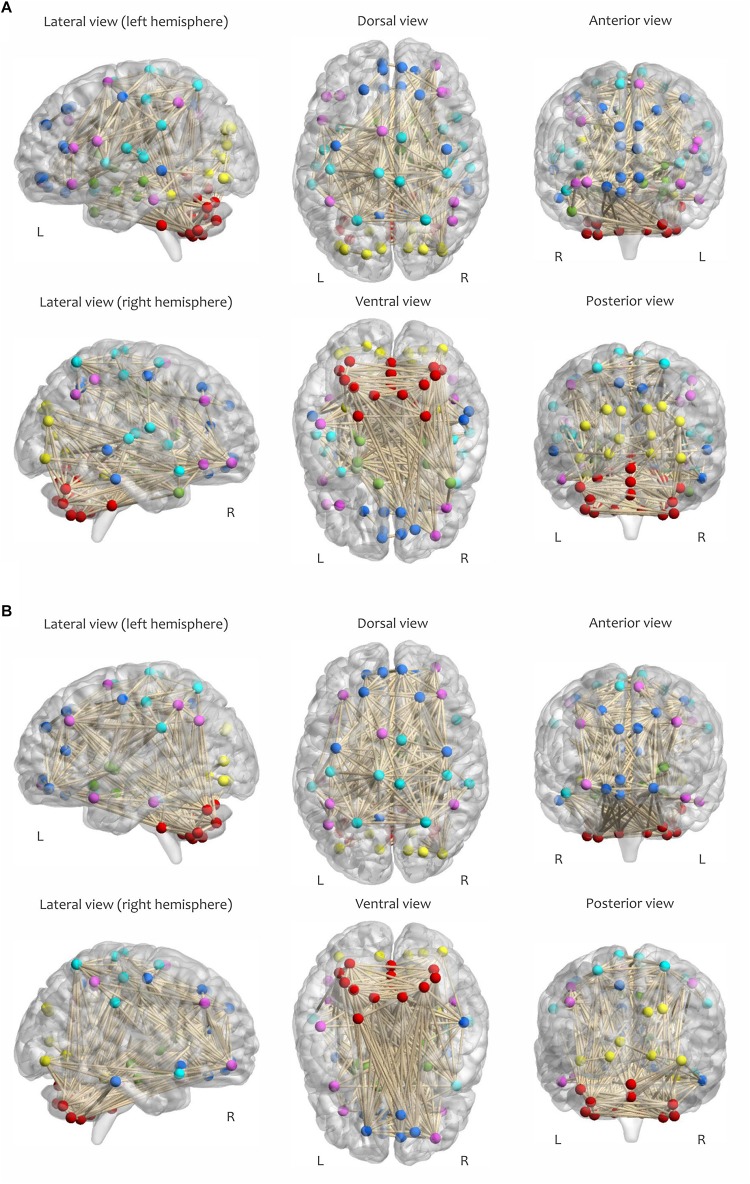
Visualization of the adjacency matrix of a participant in **(A)** RW and **(B)** SR conditions at 2:00 PM, determined by maintaining the strongest connections at δ = 0.08. The sensorimotor network is cyan, the visual network is yellow, the frontoparietal network is magenta, the default mode network is blue, the subcortical/limbic system is green, and the cerebellar network is red.

The calculation of most graph measures requires sparse matrices (Wang et al., 2010; Power et al., 2011). Therefore, all correlation matrices were subsequently thresholded and binarized by maintaining a proportion of the strongest links and eliminating the weaker connections (van den Heuvel et al., 2017). This procedure yielded an adjacency matrix *A*_*ij*_ corresponding to each of the correlation matrices with (*i*, *j*)-th entry equal to 1 if *C*_*ij*_ > ρ and 0 otherwise. The proportional value of ρ for each network was chosen individually to ensure equal network density δ (which is determined as the ratio of the number of edges to the number of possible edges in a network) across all samples; this procedure is essential for comparing network properties within or between subjects (Gamboa et al., 2014). Of note, all self-connections in the binary matrices (along the diagonal) were also set to zero during this step. Figure 4 visualizes the adjacency matrix of a participant (δ = 0.08) in both RW and SR conditions, with nodes colored according to functional network membership.

Eventually, we extracted the most common global and local graph metrics of all samples across network densities, ranging from 0.06 to 0.3 with a step size of 0.01, to identify brain topological alterations between RW and SR, as well as connectivity changes over the course of the day in both conditions. The selected density range will largely preclude the formation of disconnected or densely connected networks (Miri Ashtiani et al., 2018). All graph measures in this study were calculated with the Brain Connectivity Toolbox (BCT)^2^ (Rubinov and Sporns, 2010).

#### Global and Local Measures

**FIGURE 5:**
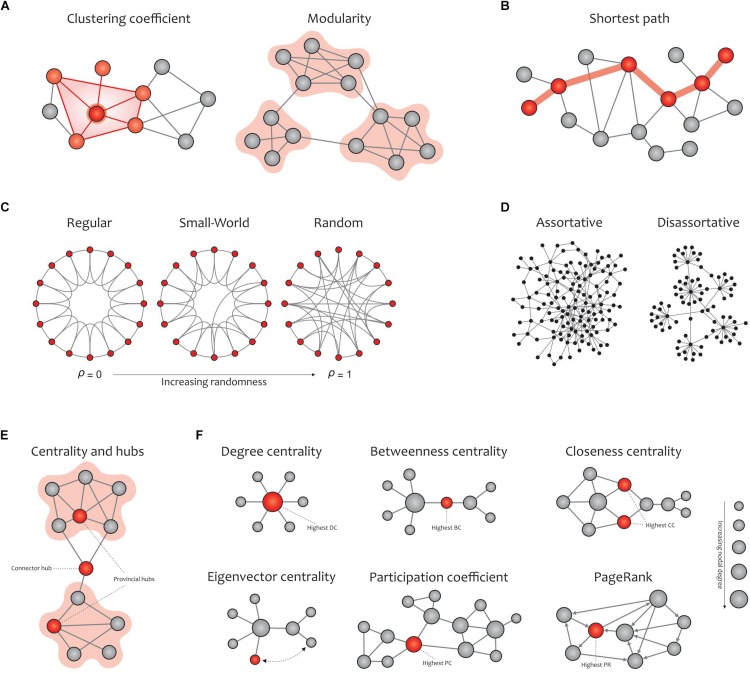
Global **(A–D)** and local **(E,F)** graph measures. **(A)** Segregation measures include the clustering coefficient, which computes the extent to which the neighbors of a given node are interconnected, and modularity, which reflects clusters of densely interconnected nodes with sparse connections among other clusters. **(B)** Integration measures include the characteristic path length, which quantifies the potential for information transmission and is determined as the average shortest path length between nodes. **(C)** Small-worldness is dedicated to graphs in which most nodes are not neighbors but can be reached by any other node with the minimum possible path length. Small-world networks exhibit an intermediate balance between regular and random networks (i.e., they consist of many short-range links alongside a few long-range links), thus reflecting a high clustering coefficient and a short path length. **(D)** The assortativity index measures the extent to which a network can resist failures in its main components. **(E)** Hubs refer to nodes with a high nodal centrality, which are classified as connector or provincial. **(F)** Network centrality measures: degree centrality (the number of node’s neighbors), betweenness centrality (the ratio of all shortest paths in the network that contain a given node), closeness centrality (the average of the shortest paths from a given node in a connected graph to every other nodes), eigenvector centrality (a self-referential index which computes the centrality of a node based on the centrality of its neighbors; here, the red node is more central than the gray node while their degrees are equal), participation coefficient (the distribution of a node’s connections across its communities), and PageRank (a variant of eigenvector centrality that is used by Google Search to determine a page’s importance). The size of the nodes in all cases is proportional to the node degree, and the red nodes (except in the eigenvalue centrality) are the most central with respect to the corresponding definition of centrality, even though their degrees are low. Adapted from Farahani et al. (2019).

Graph metrics can be classified into two main categories: global and local measures (Figure 5). Global measures are primarily aimed at revealing the functional *segregation* (e.g., clustering coefficient, modularity, and transitivity; Figure 5A) and *integration* (e.g., characteristic path length and global efficiency; Figure 5B) of information flow in brain networks, and were therefore computed here. The *small-world* property displays an optimal balance between network segregation and integration (Figure 5C). In addition to global descriptors of segregation and integration, we calculated *assortativity* (Figure 5D), a global metric that reflects network resilience to random or deliberate failures (Rubinov and Sporns, 2010; Bullmore and Bassett, 2011; Farahani et al., 2019).

On the other hand, local measures in human brain functional networks mainly provide insight into the *nodal centrality* and density of *hubs*. In network neuroscience, hubs (either connector or provincial; Figure 5E) are thought to play a key role in transferring signals among brain regions during resting and task states (Liang et al., 2013). Connector hubs interconnect nodes belonging to different modules, and provincial hubs are responsible for linking the nodes in the same module (He et al., 2009; Power et al., 2013). We calculated the most widely used local graph measures for evaluating the nodal centrality and detecting hubs in a network (Figure 5F), including the nodal degree, betweenness centrality, closeness centrality, participation coefficient, diversity coefficient, subgraph centrality, K-coreness centrality, PageRank centrality, and eigenvector centrality (Boccaletti et al., 2006; Rubinov and Sporns, 2010; Zuo et al., 2012).

### Statistical Analyses

Group differences of behavioral data were tested with paired *t*-tests and chi-square tests for comparing the means and variances of measures, respectively, in rested wakefulness and sleep restriction. A two-way repeated-measures analysis of variance (ANOVA) with *post hoc* test was carried out to assess the statistical significance of the effect of interest in all global and local graph properties. The two within-subjects factors were the condition (rested wakefulness and sleep restriction) and time (10:00 AM, 2:00 PM, 6:00 PM and 10:00 PM). The research questions were whether the condition, time, or interaction of these two factors affected the topological properties of the human brain that were modeled and computed as graph measures. A false discovery rate (FDR) procedure (Benjamini and Hochberg, 1995) was performed to adjust for multiple comparisons (corrected statistical threshold α = 0.05). Statistical tests were performed separately for each network density,δ, ranging from 0.06 to 0.3 with a step size of 0.01, on each of the computed graph measures of all samples (i.e., 25 tests for each measure).

## Results

### Vigilance Task

The subjects’ sustained attention was evaluated with three categories of behavioral measures: accuracy, response time (RT), and lapses. Table 1 shows the results of sustained attention performance in this study. Accuracy was defined as a ratio of the number of correct responses to the total number of trials. Response time was defined as the time difference between the appearance of the target and the beginning of the saccade (eye movements faster than 5°/s), if subjects accomplished the task correctly. Lapses represented responses with response times longer than 500 ms.

**TABLE 1:** Sustained attention performance.

**Behavioral measure**	**Rested Wakefulness**	**Sleep Restriction**
Accuracy (%)	81.76^∗^ ± 16.06	76.37^∗^ ± 16.34
Average RT (ms)	161.06 ± 19.81^∗∗^	165.24 ± 26.98^∗∗^
Number of lapses	2.00^∗^ ± 3.15	3.77^∗^ ± 3.41

*^∗^*p* < 0.05 in paired *t*-tests comparing means of measures in RW and SR. ^∗∗^*p* < 0.05 in chi-square tests comparing variances of measures in RW and SR. RT, response time; RW, rested wakefulness; SR, sleep restriction.*

### Global Graph Properties

**FIGURE 6:**
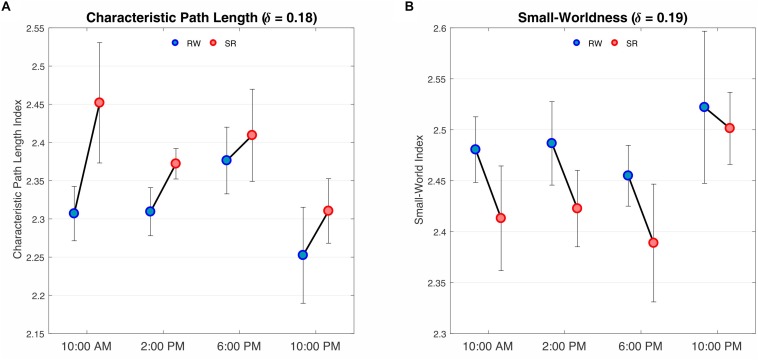
Results of two-way repeated ANOVA on the characteristic path length and small-worldness at the threshold values of 0.18 and 0.19, respectively. **(A)** The findings revealed a main effect of condition, i.e., RW versus SR (*P* < 0.05, FDR corrected), *F*(1,3) = 8.32, *P* = 0.013 but no effect of time was found. There was no interaction between condition and time (*P* > 0.05, FDR corrected). *F*(1,3) = 0.36, *P* = 0.77 Comparisons revealed that the characteristic path lengths during SR were greater than those during RW at all sampling times. **(B)** The results, as in **A**, represent a main effect of condition (*P* < 0.05, FDR corrected) *F*(1,3) = 6.84, *P* = 0.43but not time. There was no interaction between condition and time (*P* > 0.05, FDR corrected) *F*(1,3) = 0.44, *P* = 0.72. Contrast analysis showed a meaningful reduction for all periods except 10:00 PM. RW, rested wakefulness; SR, sleep restriction.

We observed a significant difference in the characteristic path length between the RW and SR conditions (*P* < 0.05 at δ = 0.17–0.19, FDR corrected), whereas the changes *p* = 0.05,δ = 0.17;*p* = 0.01,δ = 0.18;*p* = 0.02,δ = 0.19 were not significant in terms of diurnal variations or interaction (*P* > 0.05, FDR corrected). The characteristic *p* > 0.08 path length was significantly higher in SR than RW at each of the four measurement times (Figure 6A).

**FIGURE 7:**
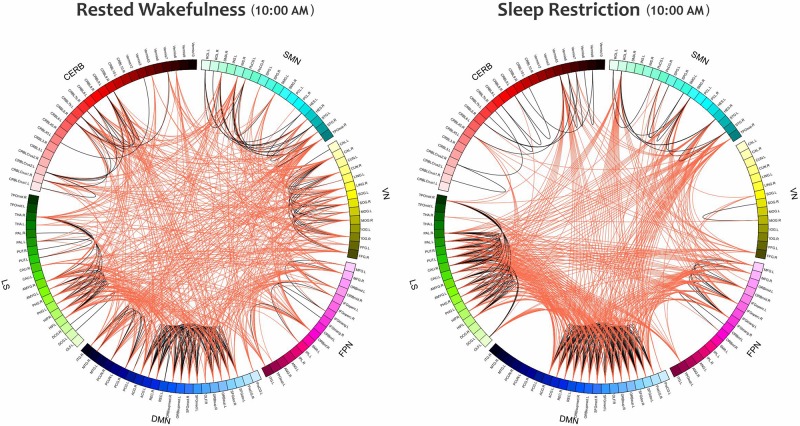
The Connectograms of a participant in both conditions at the same daily time (10:00 AM) with the thresholding value of 0.08. The **left** and **right** figures are during RW and SR, respectively. The small-world index in the RW condition was 5.21, and this value decreased to 4.02 in the SR condition, thus indicating that the network underwent topological changes. These connectograms show the functional connections within or across the sensorimotor network, visual network, frontoparietal network, default mode network, subcortical/limbic system, and cerebellum. Each brain area is represented by a square on the circumference of the external circle. The lines connecting two squares represent the functional connectivity above threshold; red lines represent inter-network connections, and black lines represent intra-network connections. This graphical representation of connectomics was created in Circos (http://circos.ca/). SMN, sensorimotor network; VN, visual network; FPN, frontoparietal network; DMN, default mode network; LS, limbic system; CERB, cerebellar network.

Furthermore, the small-world index was significantly lower under SR than RW conditions (*P* < 0.05 at δ = 0.19, FDR corrected) but there was no significant effect during the day and no significant condition-by-time interaction *p* = 0.034,δ = 0.19(*P* > 0.05, FDR corrected). *p* > 0.18Reducing the small-worldness in SR compared to RW for distinct intervals during the day is displayed in Figure 6B. Contrast analysis was meaningful for all periods except at 10:00 PM. A visual representation of the connectivity profiles between the RW and SR conditions for one participant (10:00 AM, δ = 0.08) is illustrated in Figure 7 with a connectogram framework determined in Circos software (Krzywinski et al., 2009). Parcellated regions in this graph are displayed as a circle of radially aligned elements representing the 116 brain regions in six different brain modules. The color spectrum of each module is similar to the color of the corresponding module in Figure 4, although each region is assigned a unique RGB code (raging modularly from light to dark). The red and black curves show the functional connections between and within networks, respectively. An unambiguous abbreviation scheme was created to label each parcellation, as summarized in Supplementary Table A1.

**FIGURE 8:**
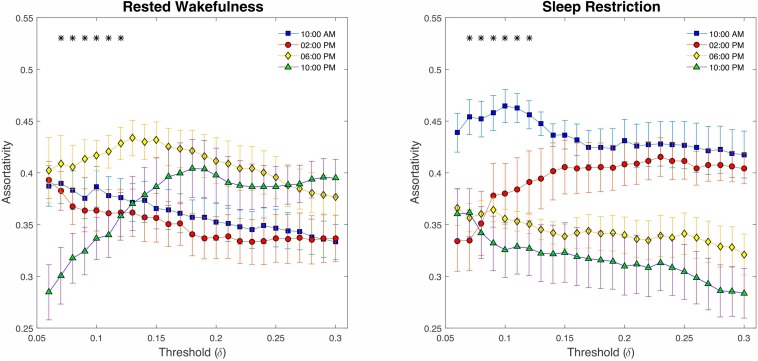
Graph assortativity analysis using fixed cost thresholds (# 25, between δ = 0.06 and δ = 0.3). At the low-range cost thresholds (0.07 < δ < 0.12), both conditions showed a significant decline across daily sampling intervals for the assortativity metric. No meaningful differences were found at the high cost thresholds (δ > 12).

Finally, examining the assortativity index (Figure 8), which represents the network’s resistance to accidental or deliberate damages to its components, demonstrated no compelling evidence of changes between SR and *p* > 0.23 RW (*P* > 0.05, FDR corrected). However, we detected a significant decline across the daily sampling intervals for this metric (*P* < 0.05 at δ = 0.07–0.12, FDR corrected).

### Local Graph Properties

**FIGURE 9:**
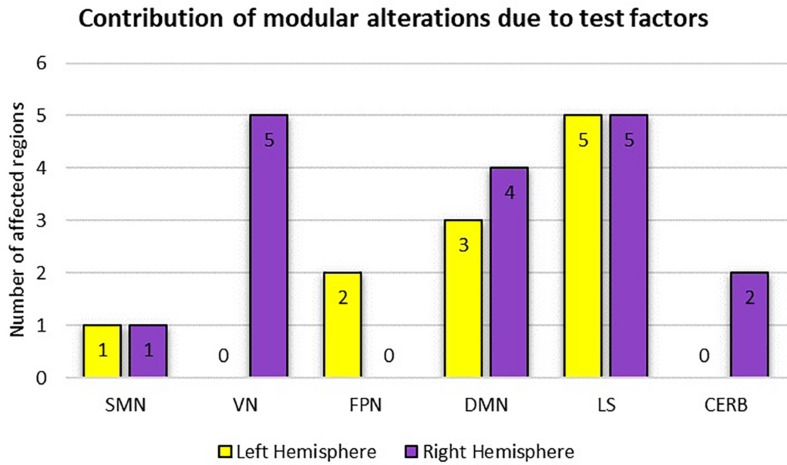
Representation of modular alterations due to the experimental factors (i.e., condition and time) with respect to both hemispheres. Here, because of the possible overlap between the two test factors, the overall effect is considered. SMN, sensorimotor network; VN, visual network; FPN, frontoparietal network; DMN, default mode network; LS, limbic system; CERB cerebellar network.

Table 2 summarizes the results of the statistical analysis for the centrality measures of the brain regions that were significant at least within one of the experimental factors (i.e., condition or time) in more than half of the binary graphs (with δ values from 0.06 to 0.3 in the step of 0.01). Numerous significant alterations were evident across the limbic system, default mode network, and visual network, whereas local measures were mostly stable in sensorimotor, frontoparietal, and cerebellar networks. As a general representation of the main effects of both experiment factors (i.e., condition and time), the number of affected areas in each of the six modules is depicted in Figure 9. The limbic system, containing the hippocampus, parahippocampal gyrus, amygdala, putamen, and globus pallidus, underwent several changes during the visual attention task in both hemispheres. Furthermore, the functional connectivity patterns within the default mode network bilaterally underwent significant alterations, particularly in the medial orbitofrontal cortex, gyrus rectus, and middle temporal gyrus. Finally, all affected regions inside the visual network were located in the right hemisphere, including the cuneus, superior occipital gyrus, middle occipital gyrus, inferior occipital cortex, and fusiform gyrus because of the participants’ right-eye dominance.

**TABLE 2:** List of brain ROIs that were significantly different within at least one of the experimental factors (i.e., condition or time) in nodal properties over more than half of the adjacency matrices (with δ values from 0.06 to 0.3 in a step of 0.01).

**ROI (modules)**	**MNI coordinates**			**AAL label**	***p*-value (δ)**						
	***x***	***y***	***z***		**Degree**	**Betweenness**	**Participation**	**K-coreness**	**Sub-graph**	**Eigenvector**	**PageRank**
9 (FPN)	−31	50	−10	Frontal_Mid_Orb_L	0.0154 (0.06)^t^						0.004 (0.09)^t^
20 (SMN)	9	0	62	Supp_Motor_Area_R	0.0063 (0.19)^t^		0.0287 (0.21)^c^	0.004 (0.23)^t^		0.0273 (0.19)^t^	
24 (DMN)	9	51	30	Frontal_Sup_Medial_R	0.0018 (0.08)^t^		0.021 (0.25)^t^	0.0038 (0.06)^t^		0.0018 (0.07)^t^	0.024 (0.06)^t^
25 (DMN)	−5	54	−7	Frontal_Med_Orb_L	0.00007 (0.1)^t^			0.00001 (0.06)^t^		0.00006 (0.28)^t^	0.00003 (0.28)^t^
26 (DMN)	8	52	−7	Frontal_Med_Orb_R	0.0008 (0.12)^t^			0.0103 (0.06)^t^		0.00037 (0.18)^t^	0.0181 (0.28)^t^
27 (DMN)	−5	37	−18	Rectus_L							0.0044 (0.09)^t^
28 (DMN)	8	36	−18	Rectus_R	0.0161 (0.17)^t^		0.0007 (0.12)^t^			0.0128 (0.17)^t^	0.0208 (0.2)^t^
34 (LS)	8	−9	40	Cingulum_Mid_R		0.0005 (0.11)^c^					
37 (LS)	−25	−21	−10	Hippocampus_L			0.0013 (0.17)^t^				
38 (LS)	29	−20	−10	Hippocampus_R		0.0073 (0.15)^c^					
39 (LS)	−21	−16	−21	ParaHippocampal_L	0.0005 (0.07)^t^			0.0002 (0.07)^t^		0.00006 (0.07)^t^	0.00006 (0.15)^t^
40 (LS)	25	−15	−20	ParaHippocampal_R	0.0264 (0.13)^t^						
41 (LS)	−23	−1	−17	Amygdala_L	0.0244 (0.3)^t^		0.0055 (0.17)^t^	0.0162 (0.23)^t^		0.0127 (0.3)^t^	
42 (LS)	27	1	−18	Amygdala_R						0.0391 (0.26)^c^	
46 (VN)	14	−79	28	Cuneus_R	0.0235 (0.06)^c^ 0.0048 (0.12)^t^		0.017 (0.25)^c^	0.0022 (0.08)^t^		0.0221 (0.1)^c^ 0.0045 (0.17)^t^	0.0189 (0.22)^c^ 0.0101 (0.12)^t^
50 (VN)	24	−81	31	Occipital_Sup_R	0.0007 (0.11)^t^			0.0004 (0.11)^t^		0.0002 (0.17)^t^	0.004 (0.19)^t^
52 (VN)	37	−80	19	Occipital_Mid_R	0.0149 (0.17)^t^			0.0103 (0.06)^t^		0.0206 (0.07)^t^	
54 (VN)	38	−82	−8	Occipital_Inf_R			0.0023 (0.11)^c^				
56 (VN)	34	−39	−20	Fusiform_R				0.0271 (0.22)^c^		0.0169 (0.13)^t^	
59 (SMN)	−23	−60	59	Parietal_Sup_L			0.0008 (0.14)^t^				
61 (FPN)	−43	−46	47	Parietal_Inf_L	0.0066 (0.16)^t^			0.0141 (0.15)^t^		0.022 (0.19)^t^	0.0082 (0.19)^t^
73 (LS)	−24	4	2	Putamen_L						0.0231 (0.3)^c^	
74 (LS)	28	5	2	Putamen_R	0.0059 (0.16)^t^			0.0118 (0.07)^t^		0.0093 (0.14)^t^	0.0048 (0.07)^t^
75 (LS)	−18	0	0	Pallidum_L	0.02 (0.3)^c^		0.0108 (0.13)^c^			0.0199 (0.3)^c^	0.0355 (0.15)^c^
85 (DMN)	−56	−34	−2	Temporal_Mid_L					0.0395 (0.21)^c^		
86 (DMN)	57	−37	−1	Temporal_Mid_R			0.0024 (0.15)^t^				
100 (CERB)	26	−58	−24	Cerebelum_6_R	0.0049 (0.13)^t^					0.0048 (0.14)^t^	0.0076 (0.13)^t^
108 (CERB)	27	−34	−41	Cerebelum_10_R	0.025 (0.18)^t^		0.0015 (0.19)^c^		0.0322 (0.23)^c^		0.0132 (0.12)^t^

*The minimum FDR adjusted *p*-value at its corresponding threshold is specified for each local feature. The superscripts *c* and *t* represent the condition and time factor, respectively. MNI, Montreal Neurological Institute space; AAL, Automated Anatomical Labeling atlas; SMN, sensorimotor network; VN, visual network; FPN, frontoparietal network; DMN, default mode network; LS, limbic system; CERB, cerebellar network.*

According to the adjusted *p*-values in Table 2, the most topological alterations between RW and SR conditions (denoted by superscript *c*) were observed in the right supplementary motor area, right midcingulate area, right hippocampus, right amygdala, right cuneus, left fusiform gyrus, left putamen, left globus pallidus, left middle temporal gyrus, and right cerebellar hemisphere (lobule 10). In contrast, we detected the brain regions of the right supplementary motor area, right medial frontal gyrus, medial orbitofrontal cortex, right gyrus rectus, left parahippocampal gyrus, left amygdala, right cuneus, right superior occipital gyrus, right middle occipital gyrus, left inferior parietal lobule, right putamen, right middle temporal gyrus, and right cerebellar hemisphere (lobules 6, 10), which changed functionally throughout the day in most local graph measures (denoted by superscript *t*).

Notably, there were no regions with closeness and diversity centrality metrics for which changes were statistically significant in more than half of the thresholded adjacency graphs. Furthermore, the betweenness and sub-graph measures were less susceptible to the brain topological alterations than the rest of the centrality measures comprising nodal degree, participation, K-coreness, eigenvector, and PageRank centrality.

**FIGURE 10:**
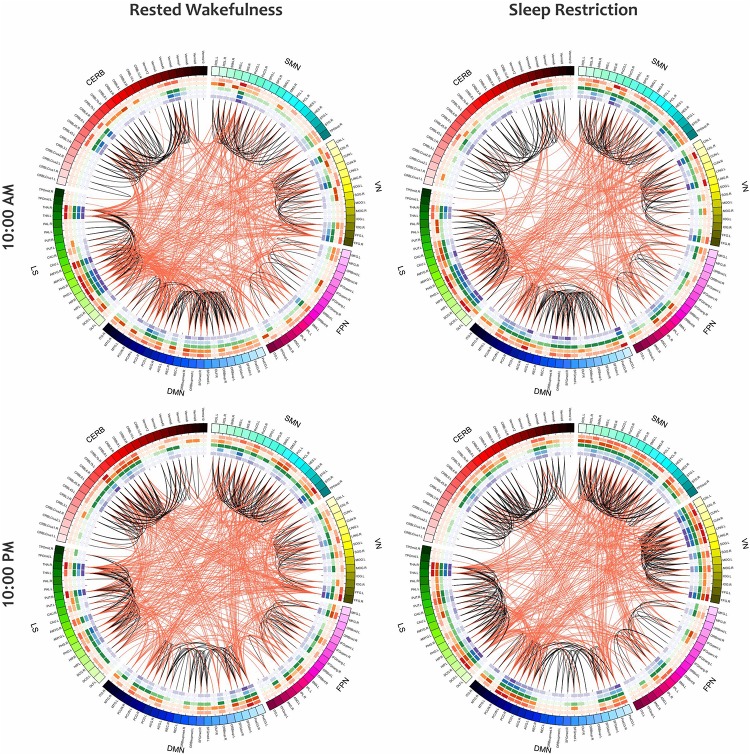
The mean connectogram for all participants under four different treatments (i.e., RW/10:00AM, RW/10:00PM, SR/10:00AM, and SR/10:00PM). Parcellated areas on the outermost circle represent the 116 AAL brain regions. This outer circle circumscribes five inner circular heat maps built to display the values of five local metrics. The range for each of these measures is from the minimum to maximum assumed value. Toward the center of the circle, these measures are degree centrality, participation coefficient, K-coreness centrality, eigenvector centrality, and PageRank. The values of all measures, as well as the functional connections in each of the connectograms, are derived from the average of all individuals in the corresponding treatment.

As a visual representation of the local properties of the functional networks in the group-level analysis, Figure 10 depicts the mean connectogram for all 13 participants in four different treatments (i.e., RW/10:00AM, RW/10:00PM, SR/10:00AM, and SR/10:00PM) at the thresholding value of 0.08. Within the outermost circle, which represents the brain parcellations, five circular heat maps were created to display five local measures associated with the corresponding parcellation. Proceeding toward the center of the circle, the measures are degree centrality, participation coefficient, K-coreness centrality, eigenvector centrality, and PageRank. The value of each local measure is indicated with a color scheme mapping that ranged from the minimum to the maximum of the data set. The values of local measures are the results of averaging these measures across all individuals. Besides, in constructing the connections in each connectogram, the correlation matrices of all participants in the corresponding treatment were first averaged, and then the result matrix was binarized with the threshold value of 0.08.

## Discussion

To the best of our knowledge, this is the first task-based fMRI study to assess whole-brain connectivity alterations after sleep curtailment, as well as diurnal variability by using graph-theoretic measures. Our study revealed two main findings in relation to global and local criteria, respectively: (1) characteristic path length, small-worldness, and assortativity were significantly altered as a result of sleep deficit or diurnal variability, however, neither the sleep condition nor the time interval in the course of a day had a main effect at the significance level of 0.05 on the values of global efficiency, local efficiency, clustering coefficient, transitivity, and modularity; (2) local graph metrics were shifted mostly across the limbic system (particularly in the hippocampus, parahippocampal gyrus, and amygdala), default mode network, and visual network, however, they were primarily stable in the sensorimotor, frontoparietal, and cerebellar networks.

As a measure of global integration, the growth of the characteristic path length in SR reflects the inefficiency of global information transfer in the brain architecture when the body experiences a lack of sleep. Moreover, as in previous research (Ferri et al., 2008), our findings revealed that brain network exhibits the small-world property during sufficient sleep and sleep-deprived conditions, however, the values in sleep-deprived mode were significantly lower than those in normal wakefulness, which contradicts the resting-state results obtained by Liu et al. (2014). According to Bassett and Bullmore (2006), the high clustering coefficient and short path length in small-world networks result in a balance between minimizing the wiring cost and maximizing the information flow among network components, indicating local specialization and global integration in the brain organization at the same time (Watts and Strogatz, 1998; Rubinov and Sporns, 2010). Therefore, the lower values of small-worldness in sleep deficit condition, as compared with wakefulness, during the spatial cueing task tend to exhibit a less optimal network topology and greater wiring cost. Eventually, our results revealed a decrease in network assortativity over the course of the day, which overlaps somewhat with the findings of Li et al. (2018). Notably, reduced assortativity is associated with a diminished tendency for a node to link to other nodes with the same or similar degree (Newman, 2003; Foster et al., 2010), thus decreasing the likelihood of a nearby hub rescuing the faulty node. Essentially, assortativity is a measure of network fault tolerance. Hence, based on our findings, brain performance appears to decrease throughout the day from morning to night because of the presence of vulnerable hubs.

Densely connected nodes and network hubs strongly affect the functional integration and segregation of the brain organization, causing a loss of network flexibility when damaged. To investigate the effects of sleep deficit or diurnal rhythms on the regional properties, several centrality measures were calculated as local features for each of the 116 ROIs. Table 2 summarizes the numerous significant alterations in these local metrics. Accordingly, the limbic system, comprising the hippocampus, parahippocampal gyrus, amygdala, putamen, and globus pallidus, bilaterally experienced the most topological alterations among brain modules during our visual attention task. These findings are consistent with other fMRI-based findings suggesting that one night of sleep loss can affect the hippocampal performance in encoding memory (Yoo et al., 2007), and disturb the functional connectivity patterns of the thalamus (Yoo et al., 2007) and the amygdala (Shao et al., 2014). Our results showed that, beyond the limbic system, the connectivity profiles within the default mode network underwent significant changes in both hemispheres, especially in the medial orbitofrontal cortex, gyrus rectus (or straight gyrus), and middle temporal gyrus, a finding consistent with previous studies (Gujar et al., 2010; Sämann et al., 2010; De Havas et al., 2012; Yeo et al., 2015). Notably, all the affected areas within the visual network were located in the right hemisphere because of the participants’ right-eye dominance (Rombouts et al., 1996). Given the higher number of significant *p*-values in Table 2 associated with time factor (denoted by superscript *t*) than condition factor (denoted by superscript *c*), diurnal variations appear to have greater effects on the reconfiguration of the brain functional connectivity than sleep curtailment.

Several challenges and future directions should be acknowledged concerning the present study. First, our sample size was relatively small. Although the results were striking, the small sample size might constrain the translational value of our findings. Therefore, further studies with larger and independent samples will be required to confirm our results. Second, we used the AAL atlas to define 116 graph nodes for brain network construction. However, there is no consensus regarding which brain parcellation scheme is optimal for specifying network nodes and constructing the brain connectivity graphs (Hayasaka and Laurienti, 2010). Different atlases may result in different topological properties in the human connectome. Thus, to ensure the reliability of the analysis, the reproducibility of the primary findings can be assessed by using multiple parcellation schemes at different spatial scales, particularly those with high resolution (Stanley et al., 2013). Third, early methods for evaluating small-worldness in real-world systems sometimes have major limitations, such as misdiagnosis of regular lattices as a small-world structure, lack of attention to weighted graphs, and neglecting the variations in network density and connection strengths. Fortunately, researchers have recently made great strides in addressing these constraints by presenting novel small-world measures (Rubinov and Sporns, 2010; Telesford et al., 2011; Bolaños et al., 2013; Muldoon et al., 2016). Applying these newly introduced metrics to future connectome research may lead to widespread progress in small-world brain architecture. Fourth, several theory-driven methods have recently begun to highlight the salient role of machine learning, algorithmic optimization, and parallel computing in fMRI analysis (Cohen et al., 2017). Therefore, adoption of modern techniques, such as multivoxel pattern analysis (MVPA), convolutional neural network (CNN), and generative models, and then aligning them with graph theoretical concepts might enable a new generation of studies to transform knowledge of neural representations in complex brain networks. Finally, the importance of hippocampal replay for network integrity during sleep loss may be another fascinating future research direction (Kumaran, 2012) that may address relevant issues related to the function of the hippocampus in the absence of sleep.

## Conclusion

The present findings based on graph-theoretic measures underline the dynamic changes in functional human connectome inflicted by sleep deficit and how they deviate from daily variability. Regional time courses were extracted from each participant during a spatial cueing task at different times, and the corresponding adjacency matrices were then constructed. By examining the commonly used global and local graph measures, we detected that the characteristic path length, small-worldness, and assortativity were significantly altered as a result of sleep loss or diurnal rhythms. However, neither the condition nor the measurement time had a main effect on global efficiency, local efficiency, clustering coefficient, transitivity, or modularity. Local graph metrics were altered mostly across the limbic system, default mode network, and visual network. In contrast, they were primarily stable in the sensorimotor, frontoparietal, and cerebellar networks.

## Data availability statement

The experimental fMRI data are available with the correspondence MF, vonfrovitz@gmail.com and magda.fafrowicz@uj.edu.pl.

## Ethics statement

The studies involving human participants were reviewed and approved by Bioethics Committee at the Jagiellonian University, Poland. The patients/participants provided their written informed consent to participate in this study.

## Author Contributions

MF and TM designed laboratory experiment and supervised data collection. MF, AD, EB, and HO contributed to data collection and preparation. AD, EB, and HO contributed to data collection and preparation. FF and WK conducted data exploration and modeling. FF prepared the initial draft of the manuscript. MF, WK, and PD supervised all aspects of manuscript preparations, revisions, editing, and final content. All authors contributed to intellectual content of the manuscript.

## Conflict of Interest

The authors declare that the research was conducted in the absence of any commercial or financial relationships that could be construed as a potential conflict of interest.

## Footnotes

1 http://www.fil.ion.ucl.ac.uk/spm

2 http://www.brain-connectivity-toolbox.net/

## References

[B1] Abós A. Baggio H. C. Segura B. García-Díaz A. I. Compta Y. Martí M. J. (2017). Discriminating cognitive status in Parkinson’s disease through functional connectomics and machine learning. *Sci. Rep.* 7 1–13. 10.1038/srep45347 28349948 PMC5368610

[B2] Anderson C. Platten C. R. (2011). Sleep deprivation lowers inhibition and enhances impulsivity to negative stimuli. *Behav. Brain Res.* 217 463–466. 10.1016/j.bbr.2010.09.020 20888369

[B3] Bassett D. S. Bullmore E. (2006). Small-world brain networks. *Neuroscientist* 12 512–523. 10.1177/1073858406293182 17079517

[B4] Benjamini Y. Hochberg Y. (1995). Controlling the false discovery rate: a practical and powerful approach to multiple testing. *J. R. Stat. Soc. Ser. B* 57 289–300. 10.1111/j.2517-6161.1995.tb02031.x

[B5] Bilek E. Schafer A. Ochs E. Esslinger C. Zangl M. Plichta M. M. (2013). Application of high-frequency repetitive transcranial magnetic stimulation to the DLPFC Alters human prefrontal-hippocampal functional interaction. *J. Neurosci.* 33 7050–7056. 10.1523/JNEUROSCI.3081-12.2013 23595762 PMC6618883

[B6] Boccaletti S. Latora V. Moreno Y. Chavez M. Hwang D. U. (2006). Complex networks: structure and dynamics. *Phys. Rep.* 424 175–308. 10.1016/j.physrep.2005.10.009

[B7] Bolaños M. Bernat E. M. He B. Aviyente S. (2013). A weighted small world network measure for assessing functional connectivity. *J. Neurosci. Methods* 212 133–142. 10.1016/j.jneumeth.2012.10.004 23085279

[B8] Bosch O. G. Rihm J. S. Scheidegger M. Landolt H.-P. Stampfli P. Brakowski J. (2013). Sleep deprivation increases dorsal nexus connectivity to the dorsolateral prefrontal cortex in humans. *Proc. Natl. Acad. Sci. U.S.A.* 110 19597–19602. 10.1073/pnas.1317010110 24218598 PMC3845164

[B9] Bullmore E. Sporns O. (2009). Complex brain networks: graph theoretical analysis of structural and functional systems. *Nat. Rev. Neurosci.* 10 186–198. 10.1038/nrn2575 19190637

[B10] Bullmore E. Sporns O. (2012). The economy of brain network organization. *Nat. Rev. Neurosci.* 13 336–349. 10.1038/nrn3214 22498897

[B11] Bullmore E. T. Bassett D. S. (2011). Brain graphs: graphical models of the human brain connectome. *Annu. Rev. Clin. Psychol.* 7 113–140. 10.1146/annurev-clinpsy-040510-143934 21128784

[B12] Buysse D. J. Reynolds C. F. Monk T. H. Berman S. R. Kupfer D. J. (1989). The pittsburgh sleep quality index: a new instrument for psychiatric practice and research. *Psychiatry Res.* 28 193–213. 10.1016/0165-1781(89)90047-4 2748771

[B13] Cirelli C. Tononi G. (2008). Is sleep essential? *PLoS Biol.* 6:e216. 10.1371/journal.pbio.0060216 18752355 PMC2525690

[B14] Cohen J. D. Daw N. Engelhardt B. Hasson U. Li K. Niv Y. (2017). Computational approaches to fMRI analysis. *Nat. Neurosci.* 20 304–313. 10.1038/nn.4499 28230848 PMC5457304

[B15] Dai Z. He Y. (2014). Disrupted structural and functional brain connectomes in mild cognitive impairment and Alzheimer’s disease. *Neurosci. Bull.* 30 217–232. 10.1007/s12264-013-1421-0 24733652 PMC5562665

[B16] De Havas J. A. Parimal S. Soon C. S. Chee M. W. L. (2012). Sleep deprivation reduces default mode network connectivity and anti-correlation during rest and task performance. *Neuroimage* 59 1745–1751. 10.1016/j.neuroimage.2011.08.026 21872664

[B17] Dinges J. D. F. Pack F. Williams K. Gillen K. A. Powell J. W. Ott G. E. (1997). Sleep deprivation and stressors: evidence for elevated negative affect in response to mild stressors when sleep deprived. *Sleep* 20 267–277. 10.1037/a0026871 9231952

[B18] Farahani F. V. Karwowski W. Lighthall N. R. (2019). Application of graph theory for identifying connectivity patterns in human brain networks: a systematic review. *Front. Neurosci.* 13:585. 10.3389/fnins.2019.00585 31249501 PMC6582769

[B19] Ferrara M. De Gennaro L. (2001). How much sleep do we need? *Sleep Med. Rev.* 5 155–179. 10.1053/smrv.2000.0138 12531052

[B20] Ferri R. Rundo F. Bruni O. Terzano M. G. Stam C. J. (2008). The functional connectivity of different EEG bands moves towards small-world network organization during sleep. *Clin. Neurophysiol.* 119 2026–2036. 10.1016/j.clinph.2008.04.294 18571469

[B21] Filippi M. van den Heuvel M. P. Fornito A. He Y. Hulshoff Pol H. E. Agosta F. (2013). Assessment of system dysfunction in the brain through MRI-based connectomics. *Lancet Neurol.* 12 1189–1199. 10.1016/S1474-4422(13)70144-3 24120645

[B22] Fleischer V. Radetz A. Ciolac D. Muthuraman M. Gonzalez-Escamilla G. Zipp F. (2017). Graph theoretical framework of brain networks in multiple sclerosis: a review of concepts. *Neuroscience* 403 35–53. 10.1016/j.neuroscience.2017.10.033 29101079

[B23] Fornito A. Bullmore E. T. (2015). Connectomics: a new paradigm for understanding brain disease. *Eur. Neuropsychopharmacol.* 25 733–748. 10.1016/j.euroneuro.2014.02.011 24726580

[B24] Fornito A. Zalesky A. Pantelis C. Bullmore E. T. (2012). Schizophrenia, neuroimaging and connectomics. *Neuroimage* 62 2296–2314. 10.1016/j.neuroimage.2011.12.090 22387165

[B25] Foster J. G. Foster D. V. Grassberger P. Paczuski M. (2010). Edge direction and the structure of networks. *Proc. Natl. Acad. Sci. U.S.A.* 107 10815–10820. 10.1073/pnas.0912671107 20505119 PMC2890716

[B26] Gamaldo C. E. Shaikh A. K. McArthur J. C. (2012). The sleep-immunity relationship. *Neurol. Clin.* 30 1313–1343. 10.1016/j.ncl.2012.08.007 23099140

[B27] Gamboa O. L. Tagliazucchi E. Von Wegner F. Jurcoane A. Wahl M. Laufs H. (2014). Working memory performance of early MS patients correlates inversely with modularity increases in resting state functional connectivity networks. *Neuroimage* 94 385–395. 10.1016/j.neuroimage.2013.12.008 24361662

[B28] Goel N. Rao H. Durmer J. S. Dinges D. F. (2009). Neurocognitive consequences of sleep deprivation. *Semin. Neurol.* 29 320–329. 10.1055/s-0029-1237117.Neurocognitive 19742409 PMC3564638

[B29] Gong Q. He Y. (2015). Depression, neuroimaging and connectomics: a selective overview. *Biol. Psychiatry* 77 223–235. 10.1016/j.biopsych.2014.08.009 25444171

[B30] Gujar N. Yoo S.-S. Hu P. Walker M. P. (2010). The unrested resting brain: sleep deprivation alters activity within the default-mode network. *J. Cogn. Neurosci.* 22 1637–1648. 10.1162/jocn.2009.21331 19702469 PMC2883887

[B31] Gujar N. Yoo S.-S. Hu P. Walker M. P. (2011). Sleep deprivation amplifies reactivity of brain reward networks, biasing the appraisal of positive emotional experiences. *J. Neurosci.* 31 4466–4474. 10.1523/JNEUROSCI.3220-10.2011 21430147 PMC3086142

[B32] Hayasaka S. Laurienti P. J. (2010). Comparison of characteristics between region-and voxel-based network analyses in resting-state fMRI data. *Neuroimage* 50 499–508. 10.1016/j.neuroimage.2009.12.051 20026219 PMC2824075

[B33] He Y. Evans A. (2010). Graph theoretical modeling of brain connectivity. *Curr. Opin. Neurol.* 23 341–350. 10.1097/WCO.0b013e32833aa567 20581686

[B34] He Y. Wang J. Wang L. Chen Z. J. Yan C. Yang H. (2009). Uncovering intrinsic modular organization of spontaneous brain activity in humans. *PLoS One* 4:e5226. 10.1371/journal.pone.0005226 19381298 PMC2668183

[B35] Hojjati S. H. Ebrahimzadeh A. Khazaee A. Babajani-Feremi A. (2017). Predicting conversion from MCI to AD using resting-state fMRI, graph theoretical approach and SVM. *J. Neurosci. Methods* 282 69–80. 10.1016/j.jneumeth.2017.03.006 28286064

[B36] Jalili M. (2017). Graph theoretical analysis of Alzheimer’s disease: discrimination of AD patients from healthy subjects. *Inf. Sci.* 384 145–156. 10.1016/j.ins.2016.08.047

[B37] Johns M. W. (1991). A new method for measuring daytime sleepiness: the epworth sleepiness scale. *Sleep* 14 540–545. 10.1093/sleep/14.6.540 1798888

[B38] Joiner T. E. (2007). Sleep disturbances and suicide risk: a review of the literature. *Neuropsychiatr. Dis. Treat.* 3 735–743. 10.2147/ndt.s1248 19300608 PMC2656315

[B39] Josephs O. Turner R. Friston K. (1997). Event-related f MRI. *Hum. Brain Mapp.* 5 243–248. 10.1002/(SICI)1097-0193(1997)5:4<243::AID-HBM7>3.0.CO;2-3 20408223

[B40] Kamphuis J. Meerlo P. Koolhaas J. M. Lancel M. (2012). Poor sleep as a potential causal factor in aggression and violence. *Sleep Med.* 13 327–334. 10.1016/j.sleep.2011.12.006 22305407

[B41] Kaufmann T. Elvsåshagen T. Alnæs D. Zak N. Pedersen P. Norbom L. B. (2016). The brain functional connectome is robustly altered by lack of sleep. *Neuroimage* 127 324–332. 10.1016/j.neuroimage.2015.12.028 26712339 PMC6600874

[B42] Krause A. J. Simon E. B. Mander B. A. Greer S. M. (2017). The sleep-deprived human brain. *Nat. Rev. Neurosci.* 18 404–418. 10.1038/nrn.2017.55 28515433 PMC6143346

[B43] Krzywinski M. Schein J. Birol I. Connors J. Gascoyne R. Horsman D. (2009). Circos?: an information aesthetic for comparative genomics. *Genome Res.* 19 1639–1645. 10.1101/gr.092759.109.19 19541911 PMC2752132

[B44] Kumaran D. (2012). What representations and computations underpin the contribution of the hippocampus to generalization and inference? *Front. Hum. Neurosci.* 6:157. 10.3389/fnhum.2012.00157 22675298 PMC3366348

[B45] Li Z. Chen R. Guan M. Wang E. Qian T. Zhao C. (2018). Disrupted brain network topology in chronic insomnia disorder: a resting-state fMRI study. *Neuroimage Clin.* 18 178–185. 10.1016/j.nicl.2018.01.012 29387533 PMC5789127

[B46] Liang X. Zou Q. He Y. Yang Y. (2013). Coupling of functional connectivity and regional cerebral blood flow reveals a physiological basis for network hubs of the human brain. *Proc. Natl. Acad. Sci. U.S.A.* 110 1929–1934. 10.1073/pnas.1214900110 23319644 PMC3562840

[B47] Lim J. Dinges D. F. (2008). Sleep deprivation and vigilant attention. *Ann. N. Y. Acad. Sci.* 1129 305–322. 10.1196/annals.1417.002 18591490

[B48] Liu H. Li H. Wang Y. Lei X. (2014). Enhanced brain small-worldness after sleep deprivation: a compensatory effect. *J. Sleep Res.* 23 554–563. 10.1111/jsr.12147 24673840

[B49] Logothetis N. K. (2002). The neural basis of the blood-oxygen-level-dependent functional magnetic resonance imaging signal. *Philos. Trans. R. Soc. Lond. Ser. B Biol. Sci.* 357 1003–1037. 10.1098/rstb.2002.1114 12217171 PMC1693017

[B50] Ma N. Dinges D. F. Basner M. Rao H. (2015). How acute total sleep loss affects the attending brain: a meta-analysis of neuroimaging studies. *Sleep* 38 233–240. 10.5665/sleep.4404 25409102 PMC4288604

[B51] Meunier D. Lambiotte R. Bullmore E. T. (2010). Modular and hierarchically modular organization of brain networks. *Front. Neurosci.* 4:200 10.3389/fnins.2010.00200 PMC3000003 21151783

[B52] Minkel J. D. Banks S. Htaik O. Moreta M. C. Jones C. W. Mcglinchey E. L. (2012). Sleep deprivation and stressors: evidence for elevated negative affect in response to mild stressors when sleep deprived. *Emotion* 12 1015–1020. 10.1037/a0026871 22309720 PMC3964364

[B53] Miri Ashtiani S. N. Daliri M. R. Behnam H. Hossein-Zadeh G. A. Mehrpour M. Motamed M. R. (2018). Altered topological properties of brain networks in the early MS patients revealed by cognitive task-related fMRI and graph theory. *Biomed. Signal Process. Control* 40 385–395. 10.1016/j.bspc.2017.10.006

[B54] Muldoon S. F. Bridgeford E. W. Bassett D. S. (2016). Small-world propensity and weighted brain networks. *Sci. Rep.* 6 1–13. 10.1038/srep22057 26912196 PMC4766852

[B55] Newman M. E. J. (2003). The structure and function of complex networks. *SIAM Rev.* 45 167–256.

[B56] Oginska H. Pokorski J. (2006). Fatigue and mood correlates of sleep length in three age-social groups: school children, students, and employees. *Chronobiol. Int.* 23 1317–1328. 10.1080/07420520601089349 17190716

[B57] Pezawas L. Meyer-Lindenberg A. Drabant E. M. Verchinski B. A. Munoz K. E. Kolachana B. S. (2005). 5-HTTLPR polymorphism impacts human cingulate-amygdala interactions: a genetic susceptibility mechanism for depression. *Nat. Neurosci.* 8 828–834. 10.1038/nn1463 15880108

[B58] Posner M. I. (1980). Orienting of attention. *Q. J. Exp. Psychol.* 32 3–25. 10.1080/00335558008248231 7367577

[B59] Power J. D. Cohen A. L. Nelson S. M. Wig G. S. Barnes K. A. Church J. A. (2011). Functional network organization of the human brain. *Neuron* 72 665–678. 10.1016/j.neuron.2011.09.006 22099467 PMC3222858

[B60] Power J. D. Schlaggar B. L. Lessov-Schlaggar C. N. Petersen S. E. (2013). Evidence for hubs in human functional brain networks. *Neuron* 79 798–813. 10.1016/j.neuron.2013.07.035 23972601 PMC3838673

[B61] Reutrakul S. Van Cauter E. (2018). Sleep in fluences on obesity, insulin resistance, and risk of type 2 diabetes. *Metabolism* 84 56–66. 10.1016/j.metabol.2018.02.010 29510179

[B62] Rogers N. L. (2003). Sleep waking and neurobehavioural performance. *Front. Biosci.* 8 s1056–s1067. 10.2741/1174 12957855

[B63] Rombouts S. A. R. B. Barkhof F. Sprenger M. Valk J. Scheltens P. (1996). The functional basis of ocular dominance: functional MRI (fMRI) findings. *Neurosci. Lett.* 221 1–4. 10.1016/S0304-3940(96)13260-2 9014166

[B64] Rubinov M. Sporns O. (2010). Complex network measures of brain connectivity: uses and interpretations. *Neuroimage* 52 1059–1069. 10.1016/j.neuroimage.2009.10.003 19819337

[B65] Sämann P. G. Tully C. Spoormaker V. I. Wetter T. C. Holsboer F. Wehrle R. (2010). Increased sleep pressure reduces resting state functional connectivity. *MAGMA* 23 375–389. 10.1007/s10334-010-0213-z 20473549

[B66] Shao Y. Lei Y. Wang L. Zhai T. Jin X. Ni W. (2014). Altered resting-state amygdala functional connectivity after 36 hours of total sleep deprivation. *PLoS One* 9:e112222. 10.1371/journal.pone.0112222 25372882 PMC4221616

[B67] Stam C. J. (2014). Modern network science of neurological disorders. *Nat. Rev. Neurosci.* 15 683–695. 10.1038/nrn3801 25186238

[B68] Stanley M. L. Moussa M. N. Paolini B. M. Lyday R. G. Burdette J. H. Laurienti P. J. (2013). Defining nodes in complex brain networks. *Front. Comput. Neurosci.* 7:169. 10.3389/fncom.2013.00169 24319426 PMC3837224

[B69] Stubbs B. Wu Y. Prina A. M. Leng Y. Cosco T. D. (2016). A population study of the association between sleep disturbance and suicidal behaviour in people with mental illness. *J. Psychiatr. Res.* 82 149–154. 10.1016/j.jpsychires.2016.07.025 27501141

[B70] Telesford Q. K. Joyce K. E. Hayasaka S. Burdette J. H. Laurienti P. J. (2011). The ubiquity of small-world networks. *Brain Connect.* 1 367–375. 10.1089/brain.2011.0038 22432451 PMC3604768

[B71] Tobaldini E. Costantino G. Solbiati M. Cogliati C. Kara T. Nobili L. (2017). Sleep, sleep deprivation, autonomic nervous system and cardiovascular diseases. *Neurosci. Biobehav. Rev.* 74 321–329. 10.1016/j.neubiorev.2016.07.004 27397854

[B72] Toni I. Schluter N. D. Josephs O. Friston K. Passingham R. E. (1999). Signal-, Set- and Movement-related Activity in the Human Brain: An Event-related fMRI Study. *Cereb. Cortex* 9 35–49. 10.1093/cercor/9.1.35 10022494

[B73] Tzourio-Mazoyer N. Landeau B. Papathanassiou D. Crivello F. Etard O. Delcroix N. (2002). Automated anatomical labeling of activations in SPM using a macroscopic anatomical parcellation of the MNI MRI single-subject brain. *Neuroimage* 15 273–289. 10.1006/nimg.2001.0978 11771995

[B74] van den Heuvel M. P. de Lange S. C. Zalesky A. Seguin C. Yeo B. T. T. Schmidt R. (2017). Proportional thresholding in resting-state fMRI functional connectivity networks and consequences for patient-control connectome studies: issues and recommendations. *Neuroimage* 152 437–449. 10.1016/j.neuroimage.2017.02.005 28167349

[B75] van den Heuvel M. P. Sporns O. (2013). Network hubs in the human brain. *Trends Cogn. Sci.* 17 683–696. 10.1016/j.tics.2013.09.012 24231140

[B76] Walker M. P. Stickgold R. (2004). Sleep-dependent learning and memory consolidation. *Neuron* 44 121–133. 10.1016/j.neuron.2004.08.031 15450165

[B77] Wang J. Zuo X. He Y. Bullmore E. T. Fornito A. (2010). Graph-based network analysis of resting-state functional MRI. *Front. Syst. Neurosci.* 4:16. 10.3389/fnsys.2010.00016 20589099 PMC2893007

[B78] Watts D. J. Strogatz S. H. (1998). Collective dynamics of “small-world” networks. *Nature* 393 440–442. 10.1038/30918 9623998

[B79] Xia M. He Y. (2011). Magnetic resonance imaging and graph theoretical analysis of complex brain networks in neuropsychiatric disorders. *Brain Connect.* 1 349–365. 10.1089/brain.2011.0062 22432450

[B80] Yeo B. T. T. Tandi J. Chee M. W. L. (2015). Functional connectivity during rested wakefulness predicts vulnerability to sleep deprivation. *Neuroimage* 111 147–158. 10.1016/j.neuroimage.2015.02.018 25700949

[B81] Yoo S. S. Hu P. T. Gujar N. Jolesz F. A. Walker M. P. (2007). A deficit in the ability to form new human memories without sleep. *Nat. Neurosci.* 10 385–392. 10.1038/nn1851 17293859

[B82] Zuo X. N. Ehmke R. Mennes M. Imperati D. Castellanos F. X. Sporns O. (2012). Network centrality in the human functional connectome. *Cereb. Cortex* 22 1862–1875. 10.1093/cercor/bhr269 21968567

