# Supplementary material for: Effects of Chronic Sleep Restriction on the Brain Functional Network, as Revealed by Graph Theory
Source: Front Neurosci. 2019 Oct 11;13:1087. doi: 10.3389/fnins.2019.01087 (PMC6807652; doi:10.3389/fnins.2019.01087)
Supplement: Supplementary file 1 [file Data_Sheet_1.docx]

**Appendix**

**Table A1.** Summary of each parcellation’s abbreviation, full description, corresponding MNI coordinates, and the associated RGB code in the connectogram.

| Abbreviation | Description | MNI coordinates | RGB code |
| --- | --- | --- | --- |
| SENSORIMOTOR NETWORK (SMN) | | | |
| ROL.L | Left Rolandic operculum | -47.16, -8.48, 13.95 | 230, 255, 242 |
| ROL.R | Right Rolandic operculum | 52.65, -6.25, 14.63 | 204, 255, 230 |
| SMA.R | Right supplementary motor area | 8.62, 0.17, 61.85 | 179, 255, 217 |
| INS.L | Left insula | -35.13, 6.65, 3.44 | 153, 255, 204 |
| INS.R | Right insula | 39.02, 6.25, 2.08 | 153, 255, 221 |
| PoCG.L | Left postcentral gyrus | -42.46, -22.63, 48.92 | 128, 255, 212 |
| PoCG.R | Right postcentral gyrus | 41.43, -25.49, 52.55 | 102, 255, 204 |
| SPG.L | Left superior parietal lobule | -23.45,-59.56, 58.96 | 102, 255, 230 |
| SPG.R | Right superior parietal lobule | 26.11, -59.18, 62.06 | 77, 255, 225 |
| SMG.L | Left supramarginal gyrus | -55.79, -33.64, 30.45 | 51, 255, 221 |
| SMG.R | Right supramarginal gyrus | 57.61, -31.5, 34.48 | 51, 255, 255 |
| PCL.L | Left paracentral lobule | -7.63, -25.36, 70.07 | 25, 255, 255 |
| PCL.R | Right paracentral lobule | 7.48, -31.59, 68.09 | 0, 255, 255 |
| HES.L | Left transverse temporal gyrus | -41.99, -18.88, 9.98 | 0, 230, 230 |
| HES.R | Right transverse temporal gyrus | 45.86, -17.15, 10.41 | 0, 204, 204 |
| STG.L | Left superior temporal gyrus | -53.16, -20.68, 7.13 | 0, 179, 179 |
| STG.R | Right superior temporal gyrus | 58.15, -21.78, 6.8 | 0, 153, 153 |
| TPOsup.R | Right superior temporal pole | 48.25, 14.75, -16.86 | 0, 128, 128 |
| VISUAL NETWORK (VN) | | | |
| CAL.L | Left calcarine sulcus | -7.14, -78.67, 6.44 | 255, 255, 204 |
| CAL.R | Right calcarine sulcus | 15.99, -73.15, 9.4 | 255, 255, 179 |
| CUN.L | Left cuneus | -5.93, -80.13, 27.22 | 255, 255, 153 |
| CUN.R | Right cuneus | 13.51, -79.36, 28.23 | 255, 255, 128 |
| LING.L | Left lingual gyrus | -14.62, -67.56, -4.63 | 255, 255, 102 |
| LING.R | Right lingual gyrus | 16.29, -66.93, -3.87 | 255, 255, 51 |
| SOG.L | Left superior occipital gyrus | -16.54, -84.26, 28.17 | 255, 255, 0 |
| SOG.R | Right superior occipital gyrus | 24.29, -80.85, 30.59 | 230, 230, 0 |
| MOG.L | Left middle occipital gyrus | -32.39, -80.73, 16.11 | 204, 204, 0 |
| MOG.R | Right middle occipital gyrus | 37.39, -79.7, 19.42 | 179, 179, 0 |
| IOG.L | Left inferior occipital cortex | -36.36, -78.29, -7.84 | 153, 153, 0 |
| IOG.R | Right inferior occipital cortex | 38.16, -81.99, -7.61 | 128, 128, 0 |
| FFG.L | Left fusiform gyrus | -31.16, -40.3, -20.23 | 102, 102, 0 |
| FFG.R | Right fusiform gyrus | 33.97, -39.1, -20.18 | 77, 77, 0 |
| FRONTOPARIETAL NETWORK (FPN) | | | |
| MFG.L | Left middle frontal gyrus | -33.43, 32.73, 35.46 | 255, 204, 255 |
| MFG.R | Right middle frontal gyrus | 37.59, 33.06, 34.04 | 255, 179, 255 |
| ORBmid.L | Left middle frontal gyrus, orbital part | -30.65, 50.43, -9.62 | 255, 153, 255 |
| ORBmid.R | Right middle frontal gyrus, orbital part | 33.18, 52.59, -10.73 | 255, 153, 238 |
| IFGoperc.L | Left inferior frontal gyrus, pars opercularis | -48.43, 12.73, 19.02 | 255, 128, 234 |
| IFGoperc.R | Right inferior frontal gyrus, pars opercularis | 50.2, 14.98, 21.41 | 255, 102, 229 |
| IFGtriang.L | Left inferior frontal gyrus, pars triangularis | -45.58, 29.91, 13.99 | 255, 77, 225 |
| IFGtriang.R | Right inferior frontal gyrus, pars triangularis | 50.33, 30.16, 14.17 | 255, 51, 221 |
| ORBinf.L | Left inferior frontal gyrus, pars orbitalis | -35.98, 30.71, -12.11 | 255, 25, 217 |
| ORBinf.R | Right inferior frontal gyrus, pars orbitalis | 41.22, 32.23, -11.91 | 255, 0, 212 |
| SMA.L | Left supplementary motor area | -5.32, 4.85, 61.38 | 255, 0, 170 |
| IPL.L | Left inferior parietal lobule | -42.8, -45.82, 46.74 | 255, 0, 128 |
| IPL.R | Right inferior parietal lobule | 46.46, -46.29, 49.54 | 230, 0, 115 |
| ANG.L | Left angular gyrus | -44.14, -60.82, 35.59 | 204, 0, 102 |
| ANG.R | Right angular gyrus | 45.51, -59.98, 38.63 | 179, 0, 89 |
| TPOsup.L | Left superior temporal pole | -39.88, 15.14, -20.18 | 153, 0, 77 |
| ITG.L | Left inferior temporal gyrus | -49.77, -28.05, -23.17 | 128, 0, 64 |
| DEFAULT MODE NETWORK (DMN) | | | |
| PreCG.L | Left precentral gyrus | -38.65, -5.68, 50.94 | 204, 238, 255 |
| PreCG.R | Right precentral gyrus | 41.37, -8.21, 52.09 | 179, 229, 255 |
| SFGdor.L | Left superior frontal gyrus | -18.45, 34.81, 42.2 | 153, 221, 255 |
| SFGdor.R | Right superior frontal gyrus | 21.9, 31.12, 43.82 | 128, 212, 255 |
| ORBsup.L | Left superior frontal gyrus, orbital part | -16.56, 47.32, -13.31 | 128, 191, 255 |
| ORBsup.R | Right superior frontal gyrus, orbital part | 18.49, 48.1, -14.02 | 102, 179, 255 |
| OLF.R | Right olfactory cortex | 10.43, 15.91, -11.26 | 77, 166, 255 |
| SFGmed.L | Left medial frontal gyrus | -4.8, 49.17, 30.89 | 51, 153, 255 |
| SFGmed.R | Right medial frontal gyrus | 9.1, 50.84, 30.22 | 51, 119, 255 |
| ORBsupmed.L | Left medial orbitofrontal cortex | -5.17, 54.06, -7.4 | 25, 102, 255 |
| ORBsupmed.R | Right medial orbitofrontal cortex | 8.16, 51.67, -7.13 | 0, 85, 255 |
| REC.L | Left gyrus rectus | -5.08, 37.07, -18.14 | 0, 77, 230 |
| REC.R | Right gyrus rectus | 8.35, 35.64, -18.04 | 0, 38, 230 |
| ACG.L | Left anterior cingulate gyrus | -4.04, 35.4, 13.95 | 0, 34, 204 |
| ACG.R | Right anterior cingulate gyrus | 8.46, 37.01, 15.84 | 0, 30, 179 |
| PCG.L | Left posterior cingulate gyrus | -4.85, -42.92, 24.67 | 0, 25, 153 |
| PCG.R | Right posterior cingulate gyrus | 7.44, -41.81, 21.87 | 0, 0 , 153 |
| PCUN.L | Left precuneus | -7.24, -56.07, 48.01 | 0, 0, 128 |
| PCUN.R | Right precuneus | 9.98, -56.05, 43.77 | 0, 0, 102 |
| MTG.L | Left middle temporal gyrus | -55.52, -33.8, -2.2 | 0, 0, 77 |
| MTG.R | Right middle temporal gyrus | 57.47, -37.23, -1.47 | 0, 0, 51 |
| ITG.R | Right inferior temporal gyrus | 53.69, -31.07, -22.32 | 0, 0, 26 |
| LIMBIC SYSTEM (LS) | | | |
| OLF.L | Left olfactory cortex | -8.06, 15.05, -11.46 | 230, 255, 204 |
| DCG.L | Left midcingulate area | -5.48, -14.92, 41.57 | 217, 255, 179 |
| DCG.R | Right midcingulate area | 8.02, -8.83, 39.79 | 204, 255, 153 |
| HIP.L | Left hippocampus | -25.03, -20.74, -10.13 | 191, 255, 128 |
| HIP.R | Right hippocampus | 29.23, -19.78, -10.33 | 179, 255, 102 |
| PHG.L | Left parahippocampal gyrus | -21.17, -15.95, -20.7 | 166, 255, 77 |
| PHG.R | Right parahippocampal gyrus | 25.38, -15.15, -20.47 | 153, 255, 40 |
| AMYG.L | Left amygdala | -23.27, -0.67, -17.14 | 128, 255, 0 |
| AMYG.R | Right amygdala | 27.32, 0.64, -17.5 | 115, 230, 0 |
| CAU.L | Left caudate nucleus | -11.46, 11, 9.24 | 76, 230, 0 |
| CAU.R | Right caudate nucleus | 14.84, 12.07, 9.42 | 68, 204, 0 |
| PUT.L | Left putamen | -23.91, 3.86, 2.4 | 34, 204, 0 |
| PUT.R | Right putamen | 27.78, 4.91, 2.46 | 30, 179, 0 |
| PAL.L | Left globus pallidus | -17.75, -0.03, 0.21 | 26, 153, 0 |
| PAL.R | Right globus pallidus | 21.2, 0.18, 0.23 | 21, 128, 0 |
| THA.L | Left thalamus | -10.85, -17.56, 7.98 | 0, 128, 0 |
| THA.R | Right thalamus | 13, -17.55, 8.09 | 0, 102, 0 |
| TPOmid.L | Left middle temporal pole | -36.32, 14.59, -34.08 | 0, 77, 0 |
| TPOmid.R | Right middle temporal pole | 44.22, 14.55, -32.23 | 0, 51, 0 |
| CEREBELLAR NETWORK (CERB) | | | |
| CRBLCrus1.L | Left crus I of cerebellar hemisphere | -36.07, -66.72, -28.93 | 255, 230, 230 |
| CRBLCrus1.R | Right crus I of cerebellar hemisphere | 37.46, -67.14, -29.55 | 255, 214, 214 |
| CRBLCrus2.L | Left crus II of cerebellar hemisphere | -28.64, -73.26, -38.20 | 255, 197, 197 |
| CRBLCrus2.R | Right crus II of cerebellar hemisphere | 32.06, -69.02, -39.95 | 255, 180, 180 |
| CRBL3.L | Left lobule III of cerebellar hemisphere | -8.80, -37.22, -18.58 | 255, 163, 163 |
| CRBL3.R | Right lobule III of cerebellar hemisphere | 12.32, -34.47, -19.39 | 255, 146, 146 |
| CRBL45.L | Left lobule IV, V of cerebellar hemisphere | -15.00, -43.49, -16.93 | 255, 129, 129 |
| CRBL45.R | Right lobule IV, V of cerebellar hemisphere | 17.20, -42.86, -18.15 | 255, 112, 112 |
| CRBL6.L | Left lobule VI of cerebellar hemisphere | -23.24, -59.10, -22.13 | 255, 95, 95 |
| CRBL6.R | Right lobule VI of cerebellar hemisphere | 24.69, -58.32, -23.65 | 255, 78, 78 |
| CRBL7b.L | Left lobule VIIB of cerebellar hemisphere | -32.36, -59.82, -45.45 | 255, 61, 61 |
| CRBL7b.R | Right lobule VIIB of cerebellar hemisphere | 33.14, -63.18, -48.46 | 255, 42, 42 |
| CRBL8.L | Left lobule VIII of cerebellar hemisphere | -25.75, -54.52, -47.68 | 255, 20, 20 |
| CRBL8.R | Right lobule VIII of cerebellar hemisphere | 25.06, -56.34, -49.47 | 255, 0, 0 |
| CRBL9.L | Left lobule IX of cerebellar hemisphere | -10.95, -48.95, -45.90 | 235, 0, 0 |
| CRBL9.R | Right lobule IX of cerebellar hemisphere | 9.46, -49.50, -46.33 | 215, 0, 0 |
| CRBL10.L | Left lobule X of cerebellar hemisphere | -22.61, -33.80, -41.76 | 195, 0, 0 |
| CRBL10.R | Left lobule X of cerebellar hemisphere | 25.99, -33.84, -41.35 | 175, 0, 0 |
| Vermis12 | Lobule I, II of vermis | 0.76, -38.79, -20.05 | 155, 0, 0 |
| Vermis3 | Lobule III of vermis | 1.38, -39.93, -11.40 | 135, 0, 0 |
| Vermis45 | Lobule IV, V of vermis | 1.22, -52.36, -6.11 | 115, 0, 0 |
| Vermis6 | Lobule VI of vermis | 1.14, -67.06, -15.12 | 95, 0, 0 |
| Vermis7 | Lobule VII of vermis | 1.15, -71.93, -25.14 | 75, 0, 0 |
| Vermis8 | Lobule VIII of vermis | 1.15, -64.43, -34.08 | 55, 0, 0 |
| Vermis9 | Lobule IX of vermis | 0.86, -54.87, -34.90 | 35, 0, 0 |
| Vermis10 | Lobule X of vermis | 0.36, -45.80, -31.68 | 15, 0, 0 |
